# Assessment of Changes in the Geographical Distribution of Opioid-Related Mortality Across the United States by Opioid Type, 1999-2016

**DOI:** 10.1001/jamanetworkopen.2019.0040

**Published:** 2019-02-22

**Authors:** Mathew V. Kiang, Sanjay Basu, Jarvis Chen, Monica J. Alexander

**Affiliations:** 1 Center for Population Health Sciences, Stanford University School of Medicine, Stanford, California; 2 Center for Primary Care, Harvard Medical School, Boston, Massachusetts; 3 Department of Social and Behavioral Sciences, Harvard T.H. Chan School of Public Health, Boston, Massachusetts; 4 Department of Statistical Sciences, University of Toronto, Toronto, Ontario, Canada; 5 Department of Sociology, University of Toronto, Toronto, Ontario, Canada

## Abstract

**Importance:**

As the opioid epidemic evolves, it is vital to identify changes in the geographical distribution of opioid-related deaths, and the specific opioids to which those deaths are attributed, to ensure that federal and state public health interventions remain appropriately targeted.

**Objective:**

To identify changes in the geographical distribution of opioid-related mortality across the United States by opioid type.

**Design, Setting, and Participants:**

Cross-sectional study using joinpoint modeling and life table analysis of individual-level data from the National Center for Health Statistics on 351 630 US residents who died from opioid-related causes from January 1, 1999, to December 31, 2016, for all of the United States and the District of Columbia. The analysis was conducted from September 6 to November 23, 2018.

**Exposures:**

Deaths involving any opioid, heroin, synthetic opioids, and natural and semisynthetic opioids.

**Main Outcomes and Measures:**

Opioid-related mortality rate, annual percent change in the opioid-related mortality rate, and life expectancy lost at age 15 years by state and opioid type.

**Results:**

From 1999 to 2016, a total of 231 264 men and 120 366 women died from opioid-related causes across the whole United States. Sixty-six observations were removed owing to missing data on age; therefore, 351 564 US residents were included in this study. The mean (SD) age at death was 39.8 (12.5) years for men and was 43.5 (12.9) years from women. Opioid-related mortality rates, especially from synthetic opioids, rapidly increased in all of the eastern United States. In most states, mortality associated with natural and semisynthetic opioids (ie, prescription painkillers) remained stable. In contrast, 28 states had mortality rates from synthetic opioids that more than doubled every 2 years (ie, annual percent change, ≥41%), including 12 with high mortality rates from synthetic opioids (>10 per 100 000 people). Among these 28 states, the mortality rate from natural and semisynthetic opioids ranged from 2.0 to 18.7 per 100 000 people (with a mean mortality rate of 6.0 per 100 000 people). The District of Columbia had the fastest rate of increase in mortality from opioids, more than tripling every year since 2013 (annual percent change, 228.3%; 95% CI, 169.7%-299.6%; *P* < .001), and a high mortality rate from synthetic opioids in 2016 (18.8 per 100 000 people); the mortality rate from natural and semisynthetic opioids was 6.9 per 100 000 people. Nationally, overall opioid-related mortality resulted in 0.36 years of life expectancy lost in 2016, which was 14% higher than deaths due to firearms and 18% higher than deaths due to motor vehicle crashes; 0.17 years of the life expectancy lost was due specifically to synthetic opioids. In 2016, New Hampshire and West Virginia lost more than 1 year of life expectancy due to opioid-related mortality.

**Conclusions and Relevance:**

Opioid-related mortality, particularly mortality associated with synthetic opioids, has increased in the eastern United States. These findings indicate that policies focused on reducing opioid-related deaths may need to prioritize synthetic opioids and rapidly expanding epidemics in northeastern states and consider the potential for synthetic opioid epidemics outside of the heroin supply.

## Key Points

**Question:**

How has opioid-related mortality changed over time across the United States, and how have the types of opioids associated with these deaths changed?

**Findings:**

In this cross-sectional study of 351 564 US residents who died from opioid-related causes, the age-standardized mortality rate from opioids increased more than 2-fold every 2 years in 24 eastern states, reflecting an expansion from lower-income, rural states. The life expectancy lost at age 15 years from opioids is now greater than that lost from deaths due to firearms or motor vehicle crashes in most of the United States.

**Meaning:**

Opioid-related mortality, driven by the use of synthetic opioids, has rapidly increased in all of the eastern states.

## Teaser

This cross-sectional study uses data from the National Center for Health Statistics to identify changes in the geographical distribution of opioid-related mortality across the United States by opioid type.

## Introduction

The opioid epidemic is one of the largest public health crises facing the United States.^1^ Opioid-related deaths in the United States have increased more than 4-fold during an 18-year period, from 2.9 (95% CI, 2.8-2.9) per 100 000 people in 1999 to 13.2 (95% CI, 13.1-13.3) per 100 000 people in 2016. This increase corresponds to more than 42 000 opioid-related deaths in 2016, many of which occurred among young adults.^2^

Although opioid-related mortality has increased steadily and exponentially since 1999, the types of opioids involved, and the places and people most affected, have changed over time. Opioid-related deaths were previously thought to be concentrated in the white population, in the Appalachian and midwestern states, and particularly induced by natural and semisynthetic prescription opioids, such as oxycodone hydrochloride.^3,4,5^ However, emerging research suggests that the opioid epidemic is not a single epidemic but multiple co-occurring epidemics marked by different types of opioids and diverse geographical, temporal, and sociodemographic patterns. Specifically, research suggests the opioid epidemic has evolved as a series of 3 intertwined but distinct epidemics, or waves, based on the types of opioids associated with mortality. In the first wave, opioid-related deaths were associated with prescription painkillers from the 1990s until about 2010. From 2010 until the present, the second wave was associated with a large increase in heroin-related deaths. In the third and current wave, which started around 2013, the rapid increase is associated with illicitly manufactured synthetic opioids.^2,6,7^ The evolution has also seen a wider range of populations being affected, with the spread of the epidemic from rural to urban areas^2^ and considerable increases in opioid-related mortality observed in the black population.^6^

To combat the evolving epidemic, states have enacted policies ranging from restricting the supply of prescription opioids to expanding treatment and access to naloxone.^8,9,10,11^ To ensure that state policies are relevant and appropriately targeted, it is vital to identify changes in the geographical distribution of opioid-related deaths and the types of opioids associated with those deaths. Given the rapid evolution of the epidemic, it is also important to identify which areas have high mortality rates due to historical trends and which areas have newly established high mortality rates likely due to changing illicit opioid markets. The identification and characterization of opioid “hot spots”—in terms of both high mortality rates and increasing trends in mortality—may allow for better-targeted policies that address the current state of the epidemic and the needs of the population.

Previous research has highlighted the spatial differences in the opioid epidemic across broad year ranges. We systematically quantify the current rate of increase in opioid-related deaths by state and opioid type. We focus on the state level because this is the geographical level at which most control policies are enacted. In addition to analyzing current rates and trends in opioid-related mortality, we compare the number of years of life expectancy lost (LEL) due to opioid-related deaths with the number of years of LEL due to other external causes to contextualize the population-level effect of these trends. We additionally provide an online interactive tool to allow visualization of opioid-related mortality over time by state and opioid type.

## Methods

This study was reviewed by the Harvard T.H. Chan School of Public Health Institutional Review Board and was deemed exempt from full review because it uses retrospective, deidentified data on deceased individuals. This study followed the Strengthening the Reporting of Observational Studies in Epidemiology (STROBE) reporting guideline.

### Data Source and Study Population

We used multiple cause of death data from January 1, 1999, through December 31, 2016, from the National Center for Health Statistics,^12^ and we used corresponding population estimates (denominators for death rates) from the US Census.^13^ We identified opioid-related deaths through recommended guidelines^14^ (eAppendix 1 in the Supplement).

### Statistical Analysis

We calculated age-standardized mortality for the total resident population for all opioids, for natural and semisynthetic opioids, for heroin, and for synthetic opioids by state and for the overall nation. We standardized mortality rates by age using 5-year age groups (ie, 0-4 years, 5-9 years, …, ≥85 years) with the direct method, using the age distribution of the US standard population in 2000. We calculated the SEs and corresponding 95% CIs of the mortality rates using Poisson approximation.^8^

### Quantifying Trends

We analyzed trends in mortality rates by state and opioid type using joinpoint regression.^15^ The coefficient of each segment is expressed as the annual percent change (APC). Annual percent changes are described as “increasing” or “decreasing” only if they were statistically significantly different from zero at the *P* < .05 level. The current rate of increase or decrease is the APC of the most recent segment, regardless of the starting year. False discovery rate–adjusted *P* values (ie, *Q* values)^16^ are provided in an online interactive results viewer. Throughout the text, we present *P* values, which were more conservative than the corresponding *Q* values (eAppendix 2 in the Supplement). Further details of the joinpoint analysis are available in eAppendix 3 and the eFigure in the Supplement.

### Quantifying LEL

We estimated the number of years of LEL due to opioid overdoses by comparing all-cause and cause-deleted life tables using the Chiang method.^17^ Specifically, we estimated the implied number of years of LEL at age 15 years as the difference in life expectancy at age 15 years from the all-cause and cause-deleted life tables. For reference comparisons, we calculated the number of years of LEL at age 15 years for 2 additional external causes of death: firearms and motor vehicle crashes (eAppendix 4 in the Supplement).

Joinpoint modeling and life table analysis of individual-level data from the National Center for Health Statistics on 351 630 US residents who died from opioid-related causes from January 1, 1999, to December 31, 2016, for all of the United States and the District of Columbia were conducted from September 6 to November 23, 2018, using R, version 3.5.0^18^ and the Joinpoint Regression Program, version 4.6.0.0.^19^ Links to the reproducible code as well as an online interactive results viewer are available in eAppendix 5 in the Supplement.

## Results

**Figure 1.**
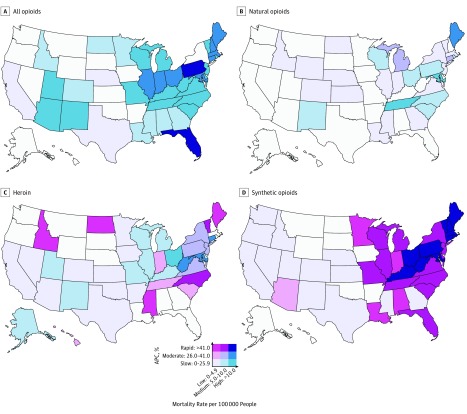
Growth and Level of the Opioid Epidemic, 2016 For each state and opioid type (A-D), we categorized the 2016 mortality rate as low (0-4.9 per 100 000 people), medium (5.0-10.0 per 100 000 people), or high (>10.0 per 100 000 people). We categorized the current annual percent change (APC) of the 2016 mortality rate as slow (0%-25.9% increase per year), moderate (26.0%-41.0% increase per year), or rapid (>41% increase per year). An annual growth rate of 26% reflects a mortality rate that is doubling every 3 years, and an annual growth rate of 41% reflects a mortality rate that is doubling every 2 years. States in white have APCs with *P* > .05. Interactive plots, which allow for specifying different breakpoints and years, are available online at https://sanjaybasu.shinyapps.io/opioid_geographic/.

From 1999 to 2016, there were more than 44.9 million deaths among US residents. We identified a total of 231 264 men and 120 366 women who died from opioid-related causes across the United States (351 630 US residents). We removed 66 observations (0.02%) owing to missing data on age (eTable 1 in the Supplement); therefore, 351 564 US residents were included in this cross-sectional study. The mean (SD) age at death was 39.8 (12.5) years for men and 43.5 (12.9) years for women. In 2016, there were 42 249 opioid-related deaths (28 498 men and 13 751 women) in the United States. This number corresponded to an age-standardized, opioid-related mortality rate of 13.2 (95% CI, 13.1-13.3) that was increasing by 18.5% (95% CI, 13.7%-23.5%; *P* < .001) per year since 2014. Higher rates of opioid-related mortality and more rapid increases in mortality were observed in the eastern United States (Figure 1). Specifically, 8 states (Connecticut, Illinois, Indiana, Massachusetts, Maryland, Maine, New Hampshire, and Ohio) had opioid-related mortality rates that were at least doubling every 3 years (APC ≥26%), and 2 states (Florida and Pennsylvania) and the District of Columbia had opioid-related mortality rates that were at least doubling every 2 years (APC ≥41%) (Figure 1). Among these 10 states and the District of Columbia with rapidly increasing mortality rates, the opioid-related mortality rate ranged from 12.6 (95% CI, 11.7-13.4) in Indiana to 35.8 (95% CI, 32.3-39.2) in New Hampshire. Only Montana and Oregon had decreasing opioid-related mortality rates (eTables 2-5 in the Supplement).

The increased mortality rates in the eastern United States were driven by synthetic opioids. Twenty-eight eastern states had mortality rates from synthetic opioids that were at least doubling every 2 years (ie, APC ≥41%), with half of those states experiencing a doubling in mortality rates from synthetic opioids every year. Among these 28 states, the mortality rate from natural and semisynthetic opioids ranged from 2.0 to 18.7 per 100 000 people (mean, 6.0 per 100 000 people). Of these 28 states, 11 (Connecticut, Kentucky, Massachusetts, Maryland, Maine, New Hampshire, Ohio, Pennsylvania, Rhode Island, Vermont, and West Virginia) and the District of Columbia had mortality rates from synthetic opioids greater than 10 per 100 000 people and had seen a rapid increase in the mortality rate, which more than doubled every 2 years (APC ≥41%) (Figure 1). Among these states with a rapid increase in mortality from opioids, the District of Columbia had a mortality rate from synthetic opioids of 18.8 (95% CI, 15.5-22.1) per 100 000 people in 2016 that was more than tripling every year (APC, 228.3%; 95% CI, 169.7%-299.6%; *P* < .001), with a mortality rate from natural and semisynthetic opioids of 6.9 per 100 000. In contrast, the national mortality rate from natural and semisynthetic opioids was lower (4.4 per 100 000 people; 95% CI, 4.3-4.5 per 100 000 people) and increased more slowly (APC, 7.4%; 95% CI, 2.3%-12.7%; *P* = .004).

**Figure 2.**
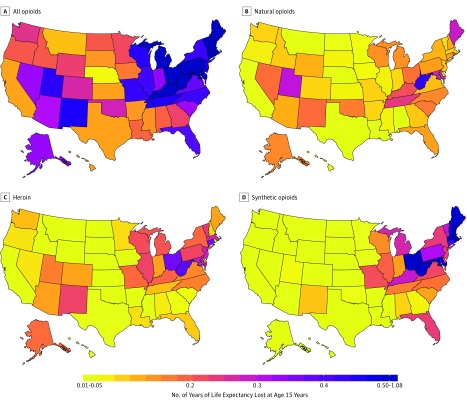
Number of Years of Life Expectancy Lost at Age 15 Years by State and Opioid Type The number of years of life expectancy lost at age 15 years is the number of life-years lost, after the age of 15 years, if all deaths from that specific cause were removed. For reference, the national number of years of life expectancy lost at age 15 years is 0.30 years for motor vehicle crashes and 0.34 years for deaths involving firearms. Additional disaggregated results for other years, ages, and reference outcomes are available online at https://sanjaybasu.shinyapps.io/opioid_geographic/.

Nationally, the number of years of LEL at age 15 years due to all fatal opioid overdoses in 2016 was 0.36 years, which was 18% higher than the number of years of LEL at age 15 years due to motor vehicle crashes (0.30 years) and 14% higher than the years of LEL at age 15 years due to firearms (0.32 years); 0.17 years of LEL was due specifically to synthetic opioids. Most states had a higher number of years of LEL at age 15 years due to opioids than due to deaths by motor vehicle accidents (N = 29 states) or firearm deaths (N = 27 states) (Table). The highest numbers for years of LEL at age 15 years were observed in the eastern states (mean, 0.49 years), while the numbers of years of LEL at age 15 years were relatively low in the western states (mean, 0.23 years) (Figure 2). In 2016 in 2 states, New Hampshire and West Virginia, the number of years of LEL at age 15 years due to opioids was greater than 1 year. The substantial geographical variation was reflected by differences in LEL from synthetic opioid deaths, which ranged from 0.02 years (Texas and Hawaii) to 0.90 years (New Hampshire).

**Table.** Hot Spots of the Opioid Epidemic, 2016^a^

State^b^	Rate (95% CI)	LEL-15^c^	APC (95% CI)	*P* Value
All opioid-related mortality				
District of Columbia	29.3 (25.2-33.3)	0.622	94.2 (10.8-240.6)	.02
Florida	14.3 (13.8-14.8)	0.397	48.2 (27.1-72.7)	<.001
Pennsylvania	18.5 (17.7-19.3)	0.523	50.1 (23.9-81.9)	<.001
Mortality from synthetic opioids				
Connecticut	14.8 (13.5-16.2)	0.424	125.0 (107.4-144.1)	<.001
District of Columbia	18.8 (15.5-22.1)	0.393	228.3 (169.7-299.6)	<.001
Kentucky	11.4 (10.4-12.5)	0.289	78.3 (56.4-103.3)	<.001
Maine	17.3 (14.9-19.8)	0.496	89.0 (62.0-120.6)	<.001
Maryland	17.7 (16.6-18.8)	0.484	128.9 (93.3-171.1)	<.001
Massachusetts	23.3 (22.1-24.5)	0.672	106.1 (74.0-144.2)	<.001
New Hampshire	30.2 (27.1-33.4)	0.904	82.6 (54.5-115.7)	<.001
Ohio	21.1 (20.3-22.0)	0.575	121.1 (98.6-146.1)	<.001
Pennsylvania	10.9 (10.3-11.5)	0.315	136.4 (111.3-164.5)	<.001
Rhode Island	17.9 (15.2-20.5)	0.507	76.5 (52.6-104.0)	<.001
Vermont	10.2 (7.4-13.0)	0.293	63.2 (35.2-97.1)	<.001
West Virginia	26.6 (24.1-29.2)	0.668	92.1 (26.8-191.0)	.005

Abbreviations: APC, annual percent change of the age-standardized rate; LEL-15, number of years of life expectancy lost at age 15 years if all deaths from that specific cause were removed.

^a^ An epidemic hot spot is a state with an age-standardized mortality rate greater than 10 per 100 000 people that is also more than doubling every 2 years (ie, APC ≥41% per year).

^b^ Additional disaggregated results for other years, ages, category cutoffs, and reference outcomes are available online at https://sanjaybasu.shinyapps.io/opioid_geographic/.

^c^ For reference, the national LEL-15 is 0.30 years for motor vehicle accidents and 0.34 y for deaths involving firearms.

Additional disaggregated results are available via the online interactive results viewer, which allows users to explore 5 sets of analyses corresponding to our results. First, we display raw opioid-related mortality rates. Second, we enable viewers to perform joinpoint analysis of epidemic hot spots. Third, we provide a national overview plot, which shows the results from all joinpoint regression models, as well as the mean change in mortality over the entire period. Fourth, for each state, we provide state-specific joinpoint results, model fit statistics, and observed rates. Finally, we allow users to view estimates of LEL at other ages, for all years, and relative to other external causes of death.

## Discussion

Although opioid-related mortality has been stereotyped as a rural, low-income phenomenon concentrated among Appalachian or midwestern states, it has spread rapidly, particularly among the eastern states. The increase in mortality has been driven primarily by synthetic opioids, which shows a distinct geographical patterning from east to west. Twenty-eight eastern states had synthetic opioid–related mortality rates that are at least doubling every 2 years, with half of those states experiencing a doubling in mortality rates every year. Of these 28 states, 12 had mortality rates from synthetic opioids greater than 10 per 100 000.

### Limitations

This study has some limitations. Our analysis assumes the accurate classification of deaths; however, opioid-related mortality may be underreported, and this underreporting may vary geographically.^20^ Data from 2014 indicate that the spatial patterning of misclassification is such that the eastern states actually have more underreporting than other states, which suggests that our results would not be driven by differential geographical underreporting.^20^ For example, in 2014, the states with the highest levels of underreporting of opioid deaths (ie, underreporting by at least 4.0 per 100 000 people) were Pennsylvania, Indiana, Louisiana, Alabama, Kentucky, Mississippi, Michigan, and Wyoming. To our knowledge, no study to date has tracked the state-level changes in underreporting over time. In addition, the presence of fentanyl requires an additional toxicology test to be requested by the coroner. Therefore, mortality rates from synthetic opioids are likely biased downward (ie, underreported), and the increase in deaths from synthetic opioids reported in recent years may be owing in part to increased detection during postmortem investigations.^21^

Despite these limitations, we believe several key points may inform health policy and public health interventions. Importantly, substantial acceleration in mortality from synthetic opioids is occurring in most eastern states regardless of past trends in deaths associated with natural and semisynthetic prescription opioids. For example, West Virginia and Kentucky, 2 states that were initial epicenters of the opioid epidemic because of high mortality rates from prescription opioids, are now experiencing rapid increases in mortality from synthetic opioids. Conversely, the District of Columbia, which did not historically have high levels of mortality from prescription opioids, has a mortality rate from synthetic opioids greater than 18 per 100 000 people and has experienced a tripling of the rate every year since 2013. By contrast, western states, such as Utah and New Mexico, that have relatively high mortality associated with prescription opioids have not experienced the same acceleration in mortality from synthetic opioids. This finding suggests that, while a “triple-wave”^2,22^ opioid epidemic has been observed at the national level and in some states, it has not been the case in all areas.

Expressing the burden of the opioid epidemic in terms of LEL highlights the fact that most opioid-related deaths are occurring among young and middle-aged adults. The results of the analysis of the numbers of years of LEL at age 15 years suggest that eliminating all deaths associated with opioid overdoses would lead to greater increases in life expectancy than would eliminating other external causes of death, such as motor vehicle crashes or deaths due to firearms. The national LEL at age 15 years due to opioids is 3 times higher than recent noted decreases in the overall life expectancy at age 15 years.^23,24^ The states with the greatest burden in terms of LEL in 2016 were no longer only West Virginia or Ohio but also included Connecticut, Maryland, Massachusetts, Rhode Island, and the District of Columbia. In 2 states, the number of years of LEL is more than 1 year. These findings are consistent with new research that suggests that the high toll of drug overdoses on life expectancy is unique to the United States among high-income countries.^25^

There is already a wide range of state policies in place to try curb the opioid epidemic. Although fatal opioid overdoses are generally increasing everywhere, there is recent evidence of decreases in some states, such as in Ohio.^26^ The multifaceted policy approach to reducing opioid-related deaths in Ohio—which includes increased access to naloxone, needle exchange programs, and increased support for those with mental health and addiction problems—may therefore serve as guidance to other states. The results presented in this article, which highlight the large heterogeneity in how the opioid epidemic has evolved across the country, suggest that policies may need to be additionally targeted, however, to take into consideration the experience of the population in terms of the prior established or unestablished nature of the opioid epidemic and the degree to which synthetic opioids are a major driver of current deaths.

## Conclusions

Opioid-related mortality, particularly mortality associated with synthetic opioids, has increased in the eastern United States. Our findings indicate that policies focused on reducing opioid-related deaths may need to prioritize synthetic opioids and rapidly expanding epidemics in northeastern states and consider the potential for synthetic opioid epidemics outside of the heroin supply.

## Notes

Supplement. **eAppendix 1.** Defining Deaths Due to Opioid Overdose, Firearms, and Car Accidents **eAppendix 2.** False Discovery Rate Adjusted *P* Values **eAppendix 3.** Details Regarding Joinpoint Models **eAppendix 4.** Estimating Implied Life Expectancy Lost **eAppendix 5.** Reproducible Code and Additional Results **eFigure.** Fitted Mortality Rate, by Opioid Type and State **eTable 1.** Descriptive Statistics of Deaths by Year and Sex **eTable 2.** Overall Opioid Mortality Rate, Annual Percent Change, and Life Expectancy Lost at Age 15 Years by State for 2016 **eTable 3.** Natural and Semisynthetic Opioid Mortality Rate, Annual Percent Change, and Life Expectancy Lost at Age 15 Years by State for 2016 **eTable 4.** Heroin Mortality Rate, Annual Percent Change, and Life Expectancy Lost at Age 15 Years by State for 2016 **eTable 5.** Synthetic Mortality Rate, Annual Percent Change, and Life Expectancy Lost at Age 15 Years by State for 2016
