# Supplementary material for: Assessment of Changes in the Geographical Distribution of Opioid-Related Mortality Across the United States by Opioid Type, 1999-2016
Source: JAMA Netw Open. 2019 Feb 22;2(2):e190040. doi: 10.1001/jamanetworkopen.2019.0040 (PMC6484620; doi:10.1001/jamanetworkopen.2019.0040)
Supplement: Supplement. — eAppendix 1. Defining Deaths Due to Opioid Overdose, Firearms, and Car Accidents eAppendix 2. False Discovery Rate Adjusted P Values eAppendix 3. Details Regarding Joinpoint Models eAppendix 4. Estimating Implied Life Expectancy Lost eAppendix 5. Reproducible Code and Additional Results eFigure. Fitted Mortality Rate, by Opioid Type and State eTable 1. Descriptive Statistics of Deaths by Year and Sex eTable 2. Overall Opioid Mortality Rate, Annual Percent Change, and Life Expectancy Lost at Age 15 Years by State for 2016 eTable 3. Natural and Semisynthetic Opioid Mortality Rate, Annual Percent Change, and Life Expectancy Lost at Age 15 Years by State for 2016 eTable 4. Heroin Mortality Rate, Annual Percent Change, and Life Expectancy Lost at Age 15 Years by State for 2016 eTable 5. Synthetic Mortality Rate, Annual Percent Change, and Life Expectancy Lost at Age 15 Years by State for 2016 [file jamanetwopen-2-e190040-s001.pdf]

Supplementary Online Content

Kiang MV, Basu S, Chen J, Alexander MJ. Assessment of changes in the geographical distribution of opioid-related mortality across the United States by opioid type, 1999-2016. *JAMA Netw Open.* 2019;2(2):e190040. doi:10.1001/jamanetworkopen.2019.0040

eAppendix 1. Defining Deaths Due to Opioid Overdose, Firearms, and Car Accidents
eAppendix 2. False Discovery Rate Adjusted *P* Values
eAppendix 3. Details Regarding Joinpoint Models
eAppendix 4. Estimating Implied Life Expectancy Lost
eAppendix 5. Reproducible Code and Additional Results
eFigure. Fitted Mortality Rate, by Opioid Type and State
eTable 1. Descriptive Statistics of Deaths by Year and Sex
eTable 2. Overall Opioid Mortality Rate, Annual Percent Change, and Life Expectancy Lost at Age 15 Years by State for 2016
eTable 3. Natural and Semisynthetic Opioid Mortality Rate, Annual Percent Change, and Life Expectancy Lost at Age 15 Years by State for 2016
eTable 4. Heroin Mortality Rate, Annual Percent Change, and Life Expectancy Lost at Age 15 Years by State for 2016
eTable 5. Synthetic Mortality Rate, Annual Percent Change, and Life Expectancy Lost at Age 15 Years by State for 2016

This supplementary material has been provided by the authors to give readers additional information about their work.

© 2019 Kiang MV et al. *JAMA Network Open.*

eAppendix 1. Defining Deaths Due to Opioid Overdose, Firearms, and Car Accidents

All opioid overdoses have one of the following underlying cause of death codes: X40-X44 (accidental), X60-X64 (self-harm), X85 (homicide), or Y10-Y14 (undetermined). In addition, opioid-related deaths also contain at least one of the following contributory cause codes: opium (T40.4), heroin (T40.1); other natural and semi-synthetic (T40.2) such as morphine, hydrocodone, or oxycodone; methadone (T40.3); other synthetic (T40.4) such as fentanyl and fentanyl derivatives; or unspecified (T40.6).

Deaths due to firearms have ICD-10 codes: *U01.4 (terrorism); W32-W34 (accidental); X72-X74 (suicide); X93-X95 (homicide); Y22-Y24 (undetermined); and Y35.0 (legal intervention). Deaths due to motor vehicle accidents have ICD-10 codes: V02–V04, V09.0, and V09.2 (pedestrian collisions); V12–V14, V19.0–V19.2, and V19.4–V19.6 (cyclist collisions); V20–V79 (motor vehicle collisions); V80.3–V80.5, V81.0–V81.1, V83–V86, V87.0, V87.8, V88.0–V88.8, V89.0, and V89.2 (other vehicle).

© 2019 Kiang MV et al. *JAMA Network Open.*

eAppendix 2. False Discovery Rate Adjusted *P* Values

Due to multiple comparisons, some significant findings may be observed due to chance alone. We use false discovery rate adjusted P-values, called Q-values, to assess this possibility. We present all P- and Q-values on the online interactive results viewer. However, in the manuscript we present only the P-values because for all levels of significance and for all types of comparisons, P-values were more conservative (i.e., fewer tests were below the significant threshold). For example, we show sets of P- and Q-value comparisons for the overall change in mortality (AAPC) as well as each joinpoint segment (APC).

Distribution of Average Annual Percent Change (AAPC) P- and Q-values.

	<1e-04	<0.001	<0.01	<0.025	<0.05	<0.1	<1
P-Value	129	145	167	177	186	193	208
Q-Value	151	178	202	208	208	208	208
Local FDR	131	148	171	184	193	198	208

Distribution of Annual Percent Change (APC) P- and Q-values.

	<1e-04	<0.001	<0.01	<0.025	<0.05	<0.1	<1
P-Value	140	197	254	275	302	330	434
Q-Value	155	222	282	325	351	378	434
Local FDR	112	161	224	246	262	284	389

On the online interactive results viewer, we present Q-values and P-values together for all results.

© 2019 Kiang MV et al. *JAMA Network Open.*

eAppendix 3. Details Regarding Joinpoint Models

Below we show the fitted mortality rate for each state (blue lines) and the national average (orange line) by opioid type from each of 208 joinpoint models. We excluded observations when there were both fewer than 100,000 people and fewer than ten deaths. In cases where an observation had fewer than ten deaths (but more than zero deaths) and a population greater than 100,000, the observation was included in the analysis but removed from the public tables and plots in accordance with National Center for Health Statistics data use requirements. The joinpoint model is log-linear, which requires all observations be non-zero and positive. Thus, in cases where there were zero deaths and a population greater than 100,000, we imputed the rate as .1 per 100,000. In addition, each observation is weighted by its precision, thus we imputed the variance of these observations with the minimum variance for that year/state/opioid type. Results were not sensitive to changes in these parameters.

eFigure shows the joinpoint-estimated opioid mortality rate, by opioid type, for each state and for the whole US. There is substantial variation in the opioid epidemic of each state and by opioid type in terms of the epidemic trajectory over time, the current mortality rate, the current growth or decline of the mortality rate, and both the timing and number of joins.

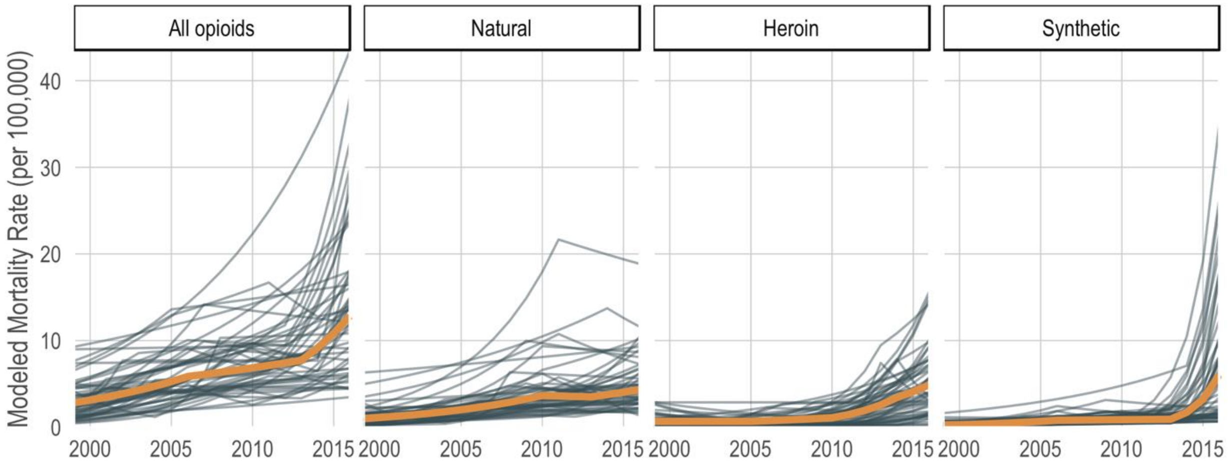

eFigure. *Fitted mortality rate, by opioid type and state. Each line represents the joinpoint model fit of opioid-related mortality. Each thin blue line is a state. The thick orange line is the national model. Note the geographic variation in trajectory, current rate of increase, and the start year of the current increases.* Interactive plots and tables, by state, are available through the interactive results viewer.

© 2019 Kiang MV et al. *JAMA Network Open.*

eAppendix 4. Estimating Implied Life Expectancy Lost

We are interested in looking at the effect of opioid overdoses on life expectancy in different populations. In order to do this, it is useful to think about mortality processes over age as a series of decrements: people can die from one of many decrements at any particular age. We want to remove one of these decrements and then see what happens to the resulting life expectancy.

We can think about different types of decrements when calculating elements of a life table. Specifically, we are interested in cause-deleted life tables: these allow you to consider mortality if one cause was completely eliminated. Cause-deleted life tables are based on the assumption that causes of death act independently, and so removing a cause does not change the hazard of dying from other causes. For causes that are often seen together (e.g., cardiovascular disease and obesity), this assumption is clearly violated. For external causes such as car accidents, firearms, and in this case, opioids, this may be a weaker assumption. To avoid issues of early life mortality, we estimate life expectancy after the age of 15. Estimates of life expectancy lost for older ages are also available on the online results viewer described in eAppendix 5.

© 2019 Kiang MV et al. *JAMA Network Open.*

eAppendix 5. Reproducible Code and Additional Results

All code to reproduce this analysis will be made available online at https://github.com/mkiang/opioid_geographic upon publication of the manuscript. While the code is freely available, the data required are restricted-access and must be requested through the National Association for Public Health Statistics and Information Systems via https://www.naphsis.org/.

We provide additional disaggregated results both through the reproducible code as well as through an online interactive results viewer available at: https://sanjaybasu.shinyapps.io/opioid_geographic/. This online interactive viewer allows users to select the outcome, year, threshold (e.g., P-value, Q-value, or minimum age for Life Expectancy Lost calculation), and state.

© 2019 Kiang MV et al. *JAMA Network Open.*

eTable 1. Descriptive Statistics of Deaths by Year and Sex

Number of observed deaths, deaths with missing age values (excluded from analysis), mean age at death, and standard deviation of age at death by year and sex.

	Male				Female			
**Year**	**Obs. (N)**	**Miss. Age (N)**	**Mean Age (y)**	**SD Age (y)**	**Obs. (N)**	**Miss. Age (N)**	**Mean Age (y)**	**SD Age (y)**
1999	5993	5	39.34	10.11	2057	1	41.30	12.10
2000	6143	9	39.08	10.30	2264	1	41.79	11.76
2001	6729	7	39.23	10.95	2767	0	41.85	11.80
2002	8160	5	39.46	11.09	3760	2	41.97	11.92
2003	8802	3	39.34	11.34	4138	0	42.43	12.11
2004	9113	2	39.12	11.83	4643	2	42.74	12.05
2005	9757	1	39.44	12.06	5161	0	43.08	12.48
2006	11600	4	39.10	12.14	5945	1	43.17	12.53
2007	11935	2	39.15	12.49	6581	0	43.41	12.64
2008	12763	1	39.36	12.55	6819	0	43.81	12.71
2009	13135	1	39.82	12.74	7287	1	44.17	12.74
2010	13355	1	39.66	12.91	7734	0	43.83	13.07
2011	14459	3	39.63	12.91	8325	1	43.68	12.89
2012	14734	2	40.23	12.95	8432	0	44.07	13.02
2013	15997	3	40.55	13.17	9055	0	44.40	13.14
2014	18420	2	40.47	13.00	10227	0	44.44	13.41
2015	21671	0	40.30	13.00	11420	2	43.74	13.41
2016	28498	2	40.00	12.90	13751	2	43.11	13.49
**All**	**231,264**	**53**	**39.77**	**12.49**	**120,366**	**13**	**43.50**	**12.91**

© 2019 Kiang MV et al. *JAMA Network Open.*

eTable 2. Overall Opioid Mortality Rate, Annual Percent Change, and Life Expectancy Lost at Age 15 Years by State for 2016

The mortality rate is expressed as deaths per 100,000 population. The Poisson approximation was used to estimate standard errors of the mortality rate. Life expectancy lost at age 15 is estimated using the Chiang method and is the difference between all-cause and cause-deleted life tables for life expectancy at age 15. Annual percent change (APC) is estimated using joinpoint regression and represents the current relative change (in percent) of the mortality rate. Additional disaggregated results for all years is available at: https://sanjaybasu.shinyapps.io/opioid_geographic/.

	**All opioids**			
**State**	**Rate (95% CI)**	**LEL_{15}**	**APC (95% CI)**	**P**
Alabama	7.5 (6.7, 8.3)	0.193	22.3 (10.3, 35.7)	0.00
Alaska	12.4 (9.8, 14.9)	0.338	0.6 (-4.7, 6.2)	0.80
Arizona	11.4 (10.5, 12.2)	0.316	13.2 (6.5, 20.2)	0.00
Arkansas	5.9 (5.0, 6.8)	0.141	1.8 (-1.1, 4.9)	0.20
California	4.8 (4.6, 5.0)	0.130	2.2 (0.6, 3.8)	0.01
Colorado	9.4 (8.6, 10.2)	0.267	5.4 (4.3, 6.5)	<.001
Connecticut	24.5 (22.8, 26.1)	0.690	30.8 (22.6, 39.5)	<.001
D.C.	29.3 (25.2, 33.3)	0.622	94.2 (10.8, 240.6)	0.02
Delaware	16.8 (14.1, 19.5)	0.474	7.2 (2.0, 12.8)	0.01
Florida	14.3 (13.8, 14.8)	0.397	48.2 (27.1, 72.7)	<.001
Georgia	8.8 (8.2, 9.3)	0.229	18.5 (12.9, 24.3)	<.001
Hawaii	5.1 (3.9, 6.3)	0.138	-0.5 (-3.2, 2.3)	0.72
Idaho	7.5 (6.1, 8.8)	0.203	4.3 (2.7, 5.9)	<.001
Illinois	15.2 (14.6, 15.9)	0.418	28.4 (0.6, 63.8)	0.05
Indiana	12.6 (11.7, 13.4)	0.340	28.2 (15.2, 42.7)	<.001
Iowa	6.2 (5.3, 7.1)	0.169	-0.1 (-3.6, 3.6)	0.97
Kansas	5.1 (4.3, 6.0)	0.133	2.8 (-0.1, 5.8)	0.06
Kentucky	23.7 (22.2, 25.2)	0.580	11.9 (10.3, 13.5)	<.001
Louisiana	7.7 (6.9, 8.5)	0.189	20.8 (12.5, 29.6)	<.001
Maine	25.4 (22.4, 28.3)	0.713	34.6 (25.4, 44.5)	<.001
Maryland	29.5 (28.1, 30.9)	0.800	30.1 (22.1, 38.6)	<.001
Massachusetts	29.6 (28.2, 30.9)	0.845	31.7 (22.7, 41.3)	<.001
Michigan	18.5 (17.7, 19.4)	0.501	24.0 (16.9, 31.5)	<.001
Minnesota	7.4 (6.6, 8.1)	0.219	5.5 (3.3, 7.9)	<.001
Mississippi	6.2 (5.2, 7.1)	0.148	22.9 (3.8, 45.6)	0.02
Missouri	15.8 (14.8, 16.9)	0.429	7.3 (5.0, 9.6)	<.001
Montana	4.1 (2.8, 5.4)	0.109	-9.2 (-14.9, -3.1)	0.01
Nebraska	2.3 (1.6, 3.1)	0.069	5.5 (2.0, 9.2)	0.00
Nevada	13.2 (11.9, 14.5)	0.334	2.9 (-5.5, 12.2)	0.45
New Hampshire	35.8 (32.3, 39.2)	1.058	34.2 (16.7, 54.3)	<.001
New Jersey	15.9 (15.0, 16.7)	0.466	21.7 (15.6, 28.1)	<.001
New Mexico	17.5 (15.6, 19.4)	0.459	4.0 (2.3, 5.6)	<.001
New York	14.8 (14.3, 15.4)	0.424	31.0 (-2.2, 75.4)	0.07
North Carolina	15.3 (14.5, 16.1)	0.419	21.9 (12.4, 32.2)	<.001
North Dakota	7.2 (5.2, 9.2)	0.203	10.1 (4.7, 15.8)	<.001
Ohio	33.0 (31.9, 34.1)	0.886	31.9 (20.3, 44.5)	<.001
Oklahoma	11.5 (10.4, 12.6)	0.275	-1.7 (-4.3, 1.0)	0.19
Oregon	7.5 (6.7, 8.4)	0.202	-2.8 (-4.8, -0.6)	0.01
Pennsylvania	18.5 (17.7, 19.3)	0.523	50.1 (23.9, 81.9)	<.001

Rhode Island	26.7 (23.5, 30.0)	0.731	9.5 (7.6, 11.4)	<.001
South Carolina	13.1 (12.0, 14.1)	0.333	12.5 (10.2, 14.8)	<.001
South Dakota	5.0 (3.5, 6.6)	0.144	1.9 (-2.6, 6.5)	0.38
Tennessee	18.1 (17.1, 19.2)	0.452	15.5 (7.5, 24.1)	0.00
Texas	4.9 (4.6, 5.1)	0.132	1.3 (0.1, 2.5)	0.03
Utah	16.4 (14.9, 18.0)	0.438	1.7 (0.6, 2.9)	0.01
Vermont	18.5 (14.8, 22.2)	0.511	16.4 (8.6, 24.8)	<.001
Virginia	13.4 (12.6, 14.2)	0.377	20.9 (5.9, 37.9)	0.01
Washington	9.3 (8.6, 10.0)	0.252	-1.0 (-2.4, 0.4)	0.13
West Virginia	43.8 (40.5, 47.0)	1.084	11.8 (8.8, 14.8)	<.001
Wisconsin	15.7 (14.6, 16.7)	0.443	9.5 (7.7, 11.4)	<.001
Wyoming	8.6 (6.2, 11.1)	0.231	1.6 (-7.7, 12.0)	0.72
**Total**	**13.2 (13.1, 13.3)**	**0.360**	**18.5 (13.7, 23.5)**	**<.001**

© 2019 Kiang MV et al. *JAMA Network Open.*

eTable 3. Natural and Semisynthetic Opioid Mortality Rate, Annual Percent Change, and Life Expectancy Lost at Age 15 Years by State for 2016

The mortality rate is expressed as deaths per 100,000 population. The Poisson approximation was used to estimate standard errors of the mortality rate. Life expectancy lost at age 15 is estimated using the Chiang method and is the difference between all-cause and cause-deleted life tables for life expectancy at age 15. Annual percent change (APC) is estimated using joinpoint regression and represents the current relative change (in percent) of the mortality rate. Additional disaggregated results for all years is available at: https://sanjaybasu.shinyapps.io/opioid_geographic/.

	Natural/semi-synthetic			
**State**	**Rate (95% CI)**	**LEL15**	**APC (95% CI)**	**P**
Alabama	2.0 (1.6, 2.4)	0.047	0.1 (-6.2, 6.8)	0.98
Alaska	6.1 (4.3, 7.9)	0.154	-1.5 (-8.3, 5.9)	0.65
Arizona	4.8 (4.3, 5.3)	0.122	-2.5 (-6.0, 1.1)	0.16
Arkansas	4.0 (3.3, 4.8)	0.094	0.0 (-3.6, 3.8)	1.00
California	2.2 (2.1, 2.4)	0.056	0.9 (-0.9, 2.9)	0.31
Colorado	3.6 (3.1, 4.1)	0.093	7.4 (5.1, 9.6)	<.001
Connecticut	5.5 (4.7, 6.3)	0.140	31.4 (17.5, 46.9)	<.001
D.C.	6.9 (5.0, 8.9)	0.144	9.6 (3.4, 16.0)	0.00
Delaware	3.4 (2.2, 4.6)	0.084	-10.9 (-24.3, 4.8)	0.15
Florida	5.1 (4.7, 5.4)	0.133	22.4 (-14.4, 75.0)	0.24
Georgia	4.3 (3.9, 4.7)	0.102	6.0 (1.9, 10.2)	0.01
Hawaii	2.8 (2.0, 3.7)	0.072	1.7 (-0.0, 3.4)	0.05
Idaho	3.9 (2.9, 4.9)	0.101	4.8 (2.9, 6.7)	<.001
Illinois	2.9 (2.6, 3.2)	0.075	22.7 (12.5, 33.8)	<.001
Indiana	3.5 (3.1, 4.0)	0.090	21.6 (4.1, 42.0)	0.02
Iowa	2.7 (2.1, 3.3)	0.069	-2.0 (-6.8, 3.1)	0.41
Kansas	2.8 (2.2, 3.4)	0.072	4.7 (2.3, 7.2)	<.001
Kentucky	9.4 (8.5, 10.4)	0.215	-1.3 (-5.8, 3.6)	0.57
Louisiana	2.3 (1.9, 2.8)	0.055	5.3 (3.0, 7.7)	<.001
Maine	10.9 (9.0, 12.8)	0.288	28.6 (15.8, 42.9)	<.001
Maryland	10.7 (9.8, 11.5)	0.276	17.2 (13.9, 20.5)	<.001
Massachusetts	3.7 (3.3, 4.2)	0.097	4.9 (1.8, 8.1)	0.00
Michigan	5.8 (5.3, 6.3)	0.148	34.6 (16.5, 55.5)	<.001
Minnesota	2.5 (2.0, 2.9)	0.066	2.4 (-2.2, 7.2)	0.28
Mississippi	3.2 (2.6, 3.9)	0.073	6.7 (1.7, 12.0)	0.01
Missouri	3.8 (3.3, 4.3)	0.097	-0.0 (-4.6, 4.8)	1.00
Montana	1.1 (0.5, 1.7)	0.025	-32.8 (-59.8, 12.5)	0.12
Nebraska	1.2 (0.7, 1.7)	0.035	5.5 (1.7, 9.5)	0.01
Nevada	7.5 (6.6, 8.5)	0.180	-7.5 (-13.6, -1.0)	0.03
New Hampshire	4.9 (3.7, 6.1)	0.126	1.5 (-5.3, 8.9)	0.64
New Jersey	3.7 (3.3, 4.1)	0.107	8.0 (5.7, 10.3)	<.001
New Mexico	7.5 (6.3, 8.7)	0.183	2.3 (0.1, 4.5)	0.04
New York	4.3 (4.0, 4.6)	0.117	7.1 (4.4, 9.7)	<.001
North Carolina	6.1 (5.6, 6.6)	0.154	14.5 (0.1, 30.9)	0.05
North Dakota	2.1 (1.1, 3.1)	0.061	8.2 (2.5, 14.2)	0.01
Ohio	7.0 (6.5, 7.5)	0.176	12.6 (10.8, 14.4)	<.001
Oklahoma	7.4 (6.5, 8.2)	0.165	-3.0 (-6.9, 1.2)	0.14
Oregon	3.0 (2.4, 3.5)	0.077	1.5 (-2.1, 5.2)	0.40
Pennsylvania	5.2 (4.7, 5.6)	0.136	12.3 (10.9, 13.7)	<.001

© 2019 Kiang MV et al. *JAMA Network Open.*

Rhode Island	8.1 (6.4, 9.8)	0.206	15.7 (12.7, 18.9)	<.001
South Carolina	6.9 (6.2, 7.7)	0.169	13.3 (11.0, 15.7)	<.001
South Dakota	2.8 (1.7, 4.0)	0.073	2.5 (-2.7, 8.1)	0.32
Tennessee	10.2 (9.4, 11.0)	0.242	7.5 (4.9, 10.2)	<.001
Texas	1.8 (1.6, 1.9)	0.044	-3.2 (-5.2, -1.1)	0.01
Utah	11.5 (10.3, 12.8)	0.299	-8.1 (-26.2, 14.3)	0.42
Vermont	3.8 (2.2, 5.5)	0.088	2.8 (0.2, 5.5)	0.04
Virginia	4.0 (3.5, 4.4)	0.108	5.8 (4.6, 7.1)	<.001
Washington	3.7 (3.3, 4.2)	0.095	-3.6 (-5.6, -1.5)	0.00
West Virginia	18.7 (16.6, 20.8)	0.432	-2.7 (-15.8, 12.3)	0.68
Wisconsin	5.7 (5.0, 6.3)	0.148	6.3 (4.3, 8.3)	<.001
Wyoming	4.3 (2.6, 6.0)	0.121	12.2 (7.3, 17.4)	<.001
**Total**	**4.4 (4.3, 4.5)**	**0.113**	**7.4 (2.3, 12.7)**	**0.01**

© 2019 Kiang MV et al. *JAMA Network Open.*

eTable 4. Heroin Mortality Rate, Annual Percent Change, and Life Expectancy Lost at Age 15 Years by State for 2016

The mortality rate is expressed as deaths per 100,000 population. The Poisson approximation was used to estimate standard errors of the mortality rate. Life expectancy lost at age 15 is estimated using the Chiang method and is the difference between all-cause and cause-deleted life tables for life expectancy at age 15. Annual percent change (APC) is estimated using joinpoint regression and represents the current relative change (in percent) of the mortality rate. Additional disaggregated results for all years is available at: https://sanjaybasu.shinyapps.io/opioid_geographic/.

	Heroin			
**State**	**Rate (95% CI)**	**LEL_{15}**	**APC (95% CI)**	**P**
Alabama	2.8 (2.3, 3.3)	0.077	6.5 (-21.5, 44.4)	0.66
Alaska	6.4 (4.6, 8.2)	0.183	23.4 (17.6, 29.5)	<.001
Arizona	4.5 (4.0, 5.0)	0.133	15.6 (12.8, 18.3)	<.001
Arkansas	0.5 (0.2, 0.7)	0.013	10.8 (5.9, 16.0)	<.001
California	1.4 (1.3, 1.5)	0.042	7.7 (4.2, 11.4)	<.001
Colorado	4.2 (3.6, 4.7)	0.131	17.9 (14.8, 21.0)	<.001
Connecticut	13.0 (11.8, 14.3)	0.375	38.6 (26.8, 51.4)	<.001
D.C.	17.1 (14.0, 20.2)	0.362	39.9 (29.8, 50.9)	<.001
Delaware	6.1 (4.5, 7.8)	0.178	25.5 (18.1, 33.4)	<.001
Florida	3.5 (3.2, 3.8)	0.101	34.5 (-2.3, 85.2)	0.06
Georgia	2.2 (1.9, 2.5)	0.065	23.7 (-5.2, 61.4)	0.11
Hawaii	1.4 (0.8, 2.1)	0.041	30.9 (13.3, 51.4)	0.00
Idaho	1.6 (1.0, 2.2)	0.052	62.8 (32.2, 100.4)	<.001
Illinois	8.2 (7.7, 8.7)	0.227	24.5 (12.8, 37.4)	<.001
Indiana	4.7 (4.2, 5.3)	0.133	30.1 (26.3, 33.9)	<.001
Iowa	1.6 (1.1, 2.1)	0.048	23.4 (19.0, 28.0)	<.001
Kansas	1.2 (0.8, 1.6)	0.033	22.0 (16.3, 27.9)	<.001
Kentucky	7.6 (6.7, 8.5)	0.192	14.2 (2.4, 27.3)	0.02
Louisiana	3.4 (2.8, 3.9)	0.087	11.0 (-6.9, 32.4)	0.21
Maine	4.7 (3.4, 6.0)	0.131	52.5 (33.5, 74.4)	<.001
Maryland	10.6 (9.8, 11.4)	0.293	39.8 (28.7, 51.8)	<.001
Massachusetts	9.4 (8.7, 10.2)	0.270	14.0 (-10.1, 44.6)	0.25
Michigan	7.6 (7.0, 8.2)	0.208	17.6 (15.4, 19.9)	<.001
Minnesota	2.8 (2.4, 3.3)	0.087	19.3 (2.8, 38.4)	0.02
Mississippi	1.2 (0.8, 1.7)	0.031	43.0 (28.0, 59.9)	<.001
Missouri	6.7 (6.0, 7.4)	0.184	11.9 (6.8, 17.3)	<.001
Montana	0.7 (0.2, 1.2)	0.026	10.9 (5.7, 16.4)	<.001
Nebraska	0.3 (0.1, 0.5)	0.009	7.7 (2.8, 12.8)	0.00
Nevada	2.9 (2.2, 3.5)	0.081	18.7 (10.0, 28.1)	<.001
New Hampshire	2.8 (1.8, 3.8)	0.088	-33.0 (-60.2, 12.8)	0.12
New Jersey	9.7 (9.0, 10.4)	0.286	35.9 (26.5, 46.0)	<.001
New Mexico	8.2 (6.9, 9.5)	0.227	12.9 (9.3, 16.7)	<.001
New York	6.5 (6.1, 6.8)	0.188	26.5 (23.5, 29.5)	<.001
North Carolina	5.7 (5.2, 6.1)	0.165	42.4 (38.2, 46.8)	<.001
North Dakota	1.5 (0.6, 2.4)	0.049	139.4 (103.8, 181.2)	<.001
Ohio	13.5 (12.8, 14.3)	0.366	14.7 (4.7, 25.6)	0.01
Oklahoma	1.4 (1.0, 1.8)	0.040	24.6 (17.0, 32.7)	<.001
Oregon	2.9 (2.3, 3.4)	0.083	1.0 (-4.3, 6.4)	0.70
Pennsylvania	7.8 (7.3, 8.3)	0.226	27.6 (23.3, 32.1)	<.001

© 2019 Kiang MV et al. *JAMA Network Open.*

Rhode Island	2.5 (1.5, 3.5)	0.074	-25.2 (-45.2, 2.0)	0.06
South Carolina	2.5 (2.1, 3.0)	0.067	39.1 (29.9, 49.0)	<.001
South Dakota	1.0 (0.3, 1.7)	0.033	106.1 (40.6, 202.1)	0.00
Tennessee	4.1 (3.6, 4.6)	0.109	33.0 (0.4, 76.1)	0.05
Texas	1.9 (1.7, 2.0)	0.056	6.8 (5.8, 7.9)	<.001
Utah	5.6 (4.7, 6.4)	0.165	11.7 (8.9, 14.6)	<.001
Vermont	8.7 (6.1, 11.2)	0.244	44.2 (33.5, 55.6)	<.001
Virginia	5.4 (4.9, 5.9)	0.155	33.0 (26.4, 40.0)	<.001
Washington	3.8 (3.4, 4.3)	0.110	-3.2 (-28.7, 31.4)	0.82
West Virginia	14.9 (13.0, 16.9)	0.390	33.8 (28.4, 39.3)	<.001
Wisconsin	7.2 (6.5, 8.0)	0.217	19.5 (12.0, 27.5)	<.001
Wyoming	1.6 (0.5, 2.6)	0.049	15.2 (8.4, 22.4)	<.001
**Total**	**4.9 (4.8, 5.0)**	**0.139**	**19.6 (10.3, 29.7)**	**0.00**

© 2019 Kiang MV et al. *JAMA Network Open.*

eTable 5. Synthetic Mortality Rate, Annual Percent Change, and Life Expectancy Lost at Age 15 Years by State for 2016

The mortality rate is expressed as deaths per 100,000 population. The Poisson approximation was used to estimate standard errors of the mortality rate. Life expectancy lost at age 15 is estimated using the Chiang method and is the difference between all-cause and cause-deleted life tables for life expectancy at age 15. Annual percent change (APC) is estimated using joinpoint regression and represents the current relative change (in percent) of the mortality rate. Additional disaggregated results for all years is available at: https://sanjaybasu.shinyapps.io/opioid_geographic/.

	Synthetic opioids			
**State**	**Rate (95% CI)**	**LEL15**	**APC (95% CI)**	**P**
Alabama	3.5 (2.9, 4.0)	0.095	105.5 (27.9, 230.1)	0.01
Alaska	1.2 (0.4, 1.9)	0.036	7.0 (-1.8, 16.7)	0.12
Arizona	1.8 (1.5, 2.1)	0.052	31.8 (15.5, 50.4)	<.001
Arkansas	1.3 (0.9, 1.8)	0.034	-6.4 (-16.7, 5.2)	0.25
California	0.9 (0.8, 1.0)	0.025	5.9 (3.5, 8.4)	<.001
Colorado	1.3 (1.0, 1.6)	0.036	6.9 (4.4, 9.5)	<.001
Connecticut	14.8 (13.5, 16.2)	0.424	125.0 (107.4, 144.1)	<.001
D.C.	18.8 (15.5, 22.1)	0.393	228.3 (169.7, 299.6)	<.001
Delaware	8.7 (6.7, 10.6)	0.253	58.9 (23.6, 104.2)	0.00
Florida	8.3 (7.9, 8.7)	0.235	116.4 (94.0, 141.5)	<.001
Georgia	2.7 (2.3, 3.0)	0.072	16.5 (12.6, 20.4)	<.001
Hawaii	0.6 (0.2, 1.0)	0.017	7.2 (1.7, 13.0)	0.01
Idaho	1.3 (0.7, 1.9)	0.034	6.3 (3.8, 8.9)	<.001
Illinois	7.1 (6.7, 7.6)	0.194	150.3 (117.3, 188.4)	<.001
Indiana	4.8 (4.3, 5.4)	0.134	103.6 (72.5, 140.2)	<.001
Iowa	2.0 (1.5, 2.5)	0.057	44.6 (-5.9, 122.1)	0.08
Kansas	1.0 (0.6, 1.3)	0.026	7.8 (4.4, 11.2)	<.001
Kentucky	11.4 (10.4, 12.5)	0.289	78.3 (56.4, 103.3)	<.001
Louisiana	2.1 (1.6, 2.5)	0.053	82.6 (25.7, 165.3)	0.00
Maine	17.3 (14.9, 19.8)	0.496	89.0 (62.0, 120.6)	<.001
Maryland	17.7 (16.6, 18.8)	0.484	128.9 (93.3, 171.1)	<.001
Massachusetts	23.3 (22.1, 24.5)	0.672	106.1 (74.0, 144.2)	<.001
Michigan	9.8 (9.2, 10.5)	0.271	136.8 (109.6, 167.5)	<.001
Minnesota	1.9 (1.5, 2.3)	0.058	62.8 (27.5, 107.8)	0.00
Mississippi	1.5 (1.1, 2.0)	0.041	19.3 (8.2, 31.6)	0.00
Missouri	7.7 (7.0, 8.5)	0.214	107.1 (8.0, 297.0)	0.03
Montana	1.4 (0.6, 2.1)	0.038	-7.7 (-15.2, 0.5)	0.06
Nebraska	0.7 (0.3, 1.1)	0.020	4.1 (0.1, 8.3)	0.04
Nevada	1.7 (1.2, 2.2)	0.041	2.4 (0.2, 4.8)	0.04
New Hampshire	30.2 (27.1, 33.4)	0.904	82.6 (54.5, 115.7)	<.001
New Jersey	7.9 (7.3, 8.5)	0.237	159.6 (121.9, 203.6)	<.001
New Mexico	4.0 (3.1, 4.9)	0.106	25.6 (10.3, 43.1)	0.00
New York	8.2 (7.8, 8.6)	0.239	140.6 (67.9, 244.9)	<.001
North Carolina	6.2 (5.7, 6.7)	0.178	71.6 (47.5, 99.7)	<.001
North Dakota	2.0 (1.0, 3.1)	0.057	5.8 (-0.1, 12.1)	0.05
Ohio	21.1 (20.3, 22.0)	0.575	121.1 (98.6, 146.1)	<.001
Oklahoma	2.5 (2.0, 3.0)	0.062	-5.5 (-10.2, -0.5)	0.04
Oregon	1.1 (0.8, 1.4)	0.029	7.4 (5.1, 9.8)	<.001
Pennsylvania	10.9 (10.3, 11.5)	0.315	136.4 (111.3, 164.5)	<.001

© 2019 Kiang MV et al. *JAMA Network Open.*

Rhode Island	17.9 (15.2, 20.5)	0.507	76.5 (52.6, 104.0)	<.001
South Carolina	5.0 (4.4, 5.7)	0.132	55.8 (29.2, 87.8)	<.001
South Dakota	1.2 (0.5, 2.0)	0.038	2.8 (-0.9, 6.5)	0.13
Tennessee	6.2 (5.5, 6.8)	0.166	69.2 (40.0, 104.4)	<.001
Texas	0.9 (0.8, 1.0)	0.023	4.8 (2.4, 7.2)	<.001
Utah	2.6 (2.0, 3.2)	0.069	8.5 (5.1, 12.0)	<.001
Vermont	10.2 (7.4, 13.0)	0.293	63.2 (35.2, 97.1)	<.001
Virginia	7.8 (7.2, 8.4)	0.223	114.5 (72.6, 166.6)	<.001
Washington	1.2 (1.0, 1.5)	0.035	4.4 (2.4, 6.5)	<.001
West Virginia	26.6 (24.1, 29.2)	0.668	92.1 (26.8, 191.0)	0.00
Wisconsin	5.3 (4.7, 5.9)	0.152	95.4 (51.0, 152.8)	<.001
Wyoming	1.1 (0.3, 1.9)	0.035	6.7 (-0.5, 14.3)	0.07
**Total**	**6.2 (6.1, 6.3)**	**0.172**	**87.6 (74.2, 102.0)**	**<.001**

© 2019 Kiang MV et al. *JAMA Network Open.*
